# Effect of rivaroxaban on preventing deep vein thrombosis in aged diabetics with femoral neck fractures after hip replacement

**DOI:** 10.1042/BSR20170289

**Published:** 2017-06-21

**Authors:** Yi-Min Zhang, Xin Jiang, Yan-Shan Sun

**Affiliations:** Department of Joint Surgery, Weifang People’s Hospital, Weifang 261000, P.R. China

**Keywords:** Diabetes, Deep-vein thrombosis, Femoral neck fractures, Hip replacement, Rivaroxaban

## Abstract

The present study estimates the effect of rivaroxaban on preventing deep vein thrombosis (DVT) in aged diabetics with femoral neck fractures after hip replacement. Our study consisted of 236 aged diabetics with femoral neck fractures, which were divided into the rivaroxaban and control groups. Reaction time (R time), clot formation time (K time), α angle (α), maximum amplitude (MA), clot elasticity (G) and coagulation index (CI), prothrombin time (PT) and activated partial thromboplastin time (APTT) were measured. DVT was diagnosed by color duplex Doppler ultrasound (CDDU). The risk factors of DVT were analysed by logistic regression analysis. Compared with the control group, in the rivaroxaban group, R time and K time were extended and α, MA and G decreased 1 day before operation. One day after operation, the rivaroxaban group had less PT and APPT and lower incidence of DVT than the control group. In the two groups, preoperative and postoperative PT and APPT significantly differed. Body mass index (BMI) ≥25, abnormal coagulation indicators, use of cemented femoral hip prosthesis, high haemoglobin content and non-ankle pump exercise after operation were the risk factors for DVT. Rivaroxaban could prevent DVT in aged diabetics with femoral neck fractures after hip replacement.

## Introduction

Diabetes, most prevalent among aged people, is considered as a disease in elder population [1]. Diabetic patients are at a higher risk of hip fracture [2]. Hip fracture affects approximately 5 million people worldwide every year and causes the loss of 2.34 million disability adjusted years of life [3]. Venous thromboembolism, also called deep vein thrombosis (DVT), is the cause of plumping, tenderness and pain in the leg, which is defined as the formation of a blot clot within a deep vein, most commonly in the leg [4]. It is a common complication after hip replacement when patients do not receive proper medical or physical prophylaxis [5]. DVT after a vascular surgery occurs at a rate of 1.7–30%, which varies with the variety and invasive degree of a surgery [6]. Venous stasis, hypercoagulability and venous valves are responsible for the formation of DVT [7,8]. Home treatment, stockings, inferior vena cava filters, thrombolysis and thrombectomy are included in the treatments of DVT [9–12]. Recent studies have shown that anticoagulation therapies with anticoagulants, such as rivaroxaban, have high efficacy and safety in the treatment of DVT [13,14].

Rivaroxaban is an oral and direct Factor Xa inhibitor, which provides a superior anticoagulation therapy in preventing and treating thromboembolic diseases [15]. Rivaroxaban is mostly used in venous thromboembolism [16,17]. With the advantages of requiring no routine coagulation monitoring and having no food interactions and little drug interaction, rivaroxaban has the same high efficacy and even higher safety than standard therapies for DVT [18]. After a comparison of rivaroxaban and enoxaparin therapies, a recent study found that rivaroxaban therapy had more advantages than disadvantages for DVT after total knee replacement and total hip replacement when compared with the enoxaparin therapy [19]. It is also reported that compared with low molecular weight heparin, anticoagulant prophylaxis using rivaroxaban after total hip or knee replacement has a lower risk of symptomatic venous thromboembolism [20]. Based on the above studies, the objective of the present study is to evaluate the effect of rivaroxaban on preventing DVT for elderly diabetic patients with femoral neck fractures undergoing hip replacement.

## Materials and methods

### Ethics statement

The present study was approved by the Medical Ethics Committee of Weifang People’s Hospital, and the medication regimen was approved by the patients and their families. All study participants provided written informed consents.

### Study subjects

From February 2011 to February 2015, 236 aged diabetic patients with femoral neck fractures admitted in Weifang People’s Hospital were included, consisting of 106 males and 130 females (mean age: 71.3 ± 4.95 years). Patients were assigned into the rivaroxaban (118 cases) and control (118 cases) groups. Inclusion criteria: patients whose weight was between 40 and 100 kg and age was more than 40 years; diabetic patients with femoral neck fractures; patients need hip replacement as a result of femoral neck fractures; patients diagnosed with negative DVT in lower limbs by preoperative color duplex Doppler ultrasound (CDDU); patients approved the medication regimen. Rejection criteria: overt bleeding tendency or contraindications to venography (allergy to contrast agent); patients with poor tolerance to rivaroxaban and heparin; patients dropped out the study or lost to follow-up. Exclusion criteria: those with weight less than 40 kg or more than 100 kg; those younger than 40 years or had a history of DVT; those with prothrombin activity lower than 60% or platelet content lower than 100 × 10^9^/l or higher than 300 × 10^9^/l; patients diagnosed with positive DVT in lower limbs by preoperative CDDU or had lower limb varicose. Hypoglycemic agents or insulin was used to regulate blood glucose levels before operation.

### Therapeutic regimens

Patients in the rivaroxaban group received oral liquid of notoginseng (10 ml/time) three times a day, Qushang tablet (3 pills/time) three times a day and rivaroxaban tablets (10 mg/time) once a day. One day before operation, these conventional therapies were discontinued and physical preventive measures, such as lifting of the affected lower limb, using of stepped elastic socks and venous pump treating for the lower limb, were conducted. Patients who were assigned to the control group were given the same therapies except for not taking rivaroxaban.

### Examination of coagulation state

A total of 3 ml venous blood were collected from each fasting patient in the morning on the admission day, 1 day before operation and 1 day after operation. Then the changes of thrombelastography (TEG) (Bayer HealthCare AG, Germany) parameters were observed: (i) reaction time (R time): the time from the beginning of examination to the amplitude of TEG reaching 2 mm, which is also the time from the blood injection into container to the coagulation, with a normal range of 12–27 min; (ii) clot formation time (K time): thrombin time, namely the time from R to the amplitude of TEG reaching 20 mm, representing the speed of clot formation, with a normal range of 3–14 min; (iii) α angle (α): the angle made by two crossed TEG curves, representing the formation speed of thrombin and faster the formation of fibrin, the greater the α would be, with a normal range of 14–46°; (iv) maximum amplitude (MA): the widest distance between the both sides of TEG, a reflection of clot strength related to fibrinogen content and platelet mass, with a normal range of 42–63 mm; (v) clot elasticity (G): normal range of 3.2–7.1 d/c and a hypercoagulable state is indicated by a G value higher than 7.1 d/c; (vi) coagulation index (CI): normal range of –3–+3, and a hypercoagulable state was represented by a CI above +3.

### Examination of coagulation function

A total of 3 ml venous blood collected from each patient on the admission day, 1 day before operation and 1 day after operation was obtained and preserved in a refrigerator (2–8°C) after centrifugation at 3000 rev/min to separate plasma. CA 1500 automatic coagulation analyzer (Sysmex, Shanghai, China) and its reagents were used to detect the preoperative and postoperative changes of haemoglobin, platelet, prothrombin time (PT) and activated partial thromboplastin time (APTT).

### Follow-up

Patients were followed up after operation until August 2016 in the forms of outpatient and telephone and 198 patients in all took part in the follow-up. The health information (sex, body mass index (BMI), history of smoking and hypertension), preoperative examination indexes (platelet counts, leucocyte counts, haemoglobin content, coagulation indicators and preoperative diagnosis of CDDU), operation indexes (hip replacement, preoperative prophylactic anticoagulation, types of prosthesis and intraoperative blood loss) and postoperative indexes (types of postoperative mechanical anticoagulation and ankle-pump exercise) of all the patients were recorded.

### Formation of DVT and occurrence of symptomatic pulmonary embolism

Two days after operation, regular CDDU was conducted to examine the formation of lower limb DVT and CT pulmonary arteriography (CTPA) was implemented in the diagnosis of symptomatic pulmonary embolism (PE). The diagnosis of DVT was performed in accordance with the diagnostic criteria proposed by Lensing et al. [21]: the DVT below the knee is called distal DVT, the DVT above the popliteal veins is called proximal DVT and the DVT extending the whole leg is called lower limb DVT. Then, the patients were assigned to the DVT and non-DVT groups.

### Occurrence of complications

The complications of patients in the rivaroxaban and control groups, for instance, slight bleeding, haematomas, subcutaneous ecchymosis, incision bleeding and major bleeding like cerebrovascular accidents and intestinal tract bleeding, were recorded 48 h after operation.

### Statistical analysis

Data were analysed by the Statistical Package for Social Science (SPSS) version 21.0 (SPSS Inc., Chicago, IL, U.S.A.). Measurement data were displayed as mean ± S.D. and the comparison between the two groups were analysed by non-paired *t* test (*P*<0.05 was considered as statistically significant) and paired *t* test was adopted to compare the different time points between groups. Enumeration data were expressed as number of cases and percentage, and the differences between the two groups were analysed by chi-square test. The risk factors of DVT were analysed by logistic regression analysis.

## Results

### Comparisons of baseline characteristics of the aged diabetic patients with femoral neck fractures between the rivaroxaban and control groups

The baseline characteristics of the subjects are shown in Table 1, which revealed no significant difference in terms of age, gender, height, injured side and Garden classification of femoral neck fracture.

**Table 1:** Comparisons of baseline characteristics of the aged diabetic patients with femoral neck fractures between the rivaroxaban and control groups

Characteristic	Control group (*n*=118)	Rivaroxaban group (*n*=118)	*P*
Age	71.9 ± 5.07	70.7 ± 4.83	0.058
Gender			0.794
Male	52	54	
Female	66	64	
Weight	62.8 ± 10.8	65.2 ± 10.1	0.079
Height	169.5 ± 6.8	168.2 ± 6.4	0.132
Injured side			0.794
Left hip	54	52	
Right hip	64	66	
Cause of injury			0.519
Trip	78	76	
Fall	26	32	
Traffic accident	14	10	
Garden types			0.624
Type I and II	8	10	
Type III	78	82	
Type IV	32	26	
Cardiac insufficiency	42	46	0.592

### Comparisons of TEG parameters between the rivaroxaban and control groups

Comparison of TEG parameters between the rivaroxaban and control groups (Table 2) showed that there was no significant difference between the two groups on the admission day. Compared with the control group, the R time extended, while α, MA and G lowered in the rivaroxaban group 1 day before operation (*P*<0.05), and there was no significant difference between K and CI (*P*>0.05). The rivaroxaban group had extended R time and K time and decreased α, MA, G and CI after operation compared with preoperative data (*P*<0.05). Compared with the control group, the rivaroxaban group had extended R time and K time and decreased α, MA, G and CI 1 day after operation (*P*<0.05). These changes indicated that rivaroxaban could lower the degree of blood hypercoagulability in diabetics.

**Table 2:** Comparisons of TEG parameters between the rivaroxaban and control groups (mean ± S.D.)

	Group	R (min)	K(min)	α (°)	MA (mm)	G (d/sc)	CI
Admission day	Control group	7.1 ± 1.2	2.5 ± 0.9	52.6 ± 14.3	70.9 ± 6.2	13.9 ± 3.2	1.4 ± 0.1
	Rivaroxaban group	7.3 ± 1.3	2.4 ± 0.5	53.1 ± 7.2	70.1 ± 6.5	13.5 ± 2.7	1.4 ± 0.1
One day before operation	Control group	7.6 ± 2.1	2.6 ± 0.4	51.6 ± 8.0	68.9 ± 6.4	11.2 ± 2.2	1.5 ± 0.2
	Rivaroxaban group	8.5 ± 1.8^†^	2.6 ± 0.3	44.5 ± 7.8^†^	56.5 ± 6.8^†^	9.0 ± 2.9^†^	1.9 ± 0.5
One day after operation	Control group	7.0 ± 1.1	2.4 ± 1.2	48.1 ± 6.5	64.6 ± 6.7	9.8 ± 1.4	1.1 ± 0.1
	Rivaroxaban group	9.3 ± 0.9^*†^	3.2 ± 1.1^*†^	35.3 ± 6.3^*†^	51.7 ± 5.4^*†^	8.3 ± 1.7^*†^	1.6 ± 0.2^*†^

*, *P*<0.05 compared with 1 day before operation; ^†^, *P*<0.05 compared with the control group.

### Comparisons of coagulation function during perioperative period between the rivaroxaban and control groups

As shown in Table 3, before operation, compared with the control group, the number of platelets and haemoglobin, as well as PT and APPT did not significantly change; after operation, compared with the control group, the number of platelets and haemoglobin was significantly lower, and the PT and APPT were significantly decreased in the rivaroxaban group (*P*<0.05). Compared with the preoperative data, there was no significant difference in PT, APPT and the number of platelets and haemoglobin in the rivaroxaban group (*P*>0.05), while in the control group, the PT and APPT extended and the number of platelets and haemoglobin increased significantly after operation (*P*<0.05).

**Table 3:** Comparison of blood coagulation function during perioperative period between the rivaroxaban and control groups

	Group	Platelet	Haemoglobin	PT	APPT
Preoperation	Control group	184.23 ± 42.71	131.56 ± 16.40	11.56 ± 0.47	30.75 ± 2.92
	Rivaroxaban group	184.18 ± 32.18	129.89 ± 17.51	11.23 ± 0.65	32.36 ± 2.54
Postoperation	Control group	195.26 ± 36.23*	137.86 ± 20.21*	13.32 ± 0.50*	34.24 ± 2.87*
	Rivaroxaban group	182.46 ± 31.27^†^	129.65 ± 18.80^†^	11.28 ± 0.64^†^	32.87 ± 2.62^†^

*, *P*<0.05 compared with preoperative data; ^†^, *P*<0.05 compared with the control group.

### Comparisons of the incidences of DVT and PE between the rivaroxaban and control groups

There were 6 patients with positive DVT among all 118 patients in the rivaroxaban group with an incidence of 5.1%, while there were 22 patients of positive DVT in 118 patients in the control group with an incidence of 18.6%, revealing a significant higher percentage of DVT in the control group than in the rivaroxaban group (22/118 compared with 6/118, *P*<0.05). There was no PE in either of the two groups (*P*<0.05).

### Comparison of general information of patients in the DVT and non-DVT groups

Univariate analysis demonstrated among the two groups that there were significant differences in BMI, heamoglobin content, abnormity of coagulation indicators, hip replacement, types of prosthesis, types of mechanical anticoagulation and ankle-pump exercises indexes between the DVT and non-DVT groups (*P*<0.05), while gender, history of smoking, hypertension, platelets, leucocyte number, preoperative lower limb vein conditions and preoperative prophylactic anticoagulation were of no significant difference (*P*>0.05) (Table 4).

**Table 4:** Comparison of general information of patients in the DVT and non-DVT groups (*n*)

Parameters	DVT group (*n*=28)	Non-DVT group (*n*=208)	χ^2^	*P*
Gender			0.962	0.327
Male	15	91		
Female	13	117		
BMI			4.602	0.032
<5	10	119		
≥25	18	89		
Smoking history			1.494	0.222
Yes	15	136		
No	13	72		
Hypertension			0.522	0.47
Yes	14	89		
No	14	119		
Platelet counts			0.029	0.864
High	15	115		
Normal	13	93		
Leucocyte counts			0.412	0.521
High	15	98		
Normal	13	110		
Haemoglobin content				
Low	8	34	16.46	0.0003
Normal	6	127		
High	14	47		
Coagulation indicators			5.754	0.017
Any exception	18	84		
Normal	10	124		
Preoperative diagnosis of CDDU			0.83	0.362
Abnormal	13	78		
Normal	15	130		
Hip replacement			5.222	0.022
One side	13	142		
Both sides	15	66		
Preoperative prophylactic anticoagulation			0.066	0.798
Yes	11	87		
No	17	121		
Types of prosthesis			6.865	0.012
Uncemented femoral hip prosthesis	11	135		
Cemented femoral hip prosthesis	17	73		
Types of mechanical anticoagulation			6.248	0.012
Elastic stockings for blood	9	119		
Circulation pump	19	89		
Ankle-pump exercises			9.208	0.002
Yes	10	136		
No	18	72		

### Logistic regression analysis for the risk factors of DVT

Postoperative occurrence of DVT was used as the dependent variable, logistic regression analysis of the indexes with *P*>0.05 in univariate analysis was performed and the results indicated that BMI ≥25, abnormal coagulation indicators, the use of cemented femoral hip prosthesis, high haemoglobin content and non-ankle pump exercises after operation were risk factors of DVT (Table 5).

**Table 5:** Logistic regression analysis for the risk factors of DVT

Factors	B	S.E.M.	Sig.	OR	95% CI
BMI ≥25	2.613	0.713	<0.001	13.642	3.376–55.134
Abnormal coagulation indicators	1.218	0.517	0.018	3.381	1.227–9.315
Mechanical anticoagulation	0.25	0.528	0.636	1.284	0.456–3.617
Use of cemented femoral hip prosthesis	1.096	0.51	0.032	2.992	1.011–8.135
Haemoglobin content	1.554	0.381	<0.001	4.732	2.242–9.984
Hip replacement	0.77	0.514	0.134	2.159	0.788–5.913
Non-ankle pump exercises after operation	1.199	0.51	0.019	3.317	1.220–9.022

Abbreviations: B, regression coefficient; OR, odds ratio; Sig., significance; 95% CI, 95% confidence interval.

### Comparisons of postoperative complications between the rivaroxaban and control groups

Postoperative complications of patients in the rivaroxaban and control groups were recorded (Table 6). It exhibited that among the 35 patients with slight bleeding in the control group, there were 10 patients with haematoma, 13 patients with subcutaneous ecchymosis and 12 patients with incision bleeding. In the rivaroxaban group, there were 12 patients with haematoma, 14 patients with subcutaneous ecchymosis and 6 patients with incision bleeding among all the 32 patients with slight bleeding. The results revealed no significant difference between the two groups (*P*>0.05). There was one case of defecate haemorrhage in the control group and zero in the rivaroxaban group, indicating no significant difference (*P*>0.05). The incision bleeding time in the control group (7.52 ± 1.32 min) was significantly higher than that in the rivaroxaban group (6.19 ± 1.31 min) (*P*<0.05).

**Table 6:** Comparisons of postoperative complications between the rivaroxaban and control groups

Complications	Control group (*n*=118)	Rivaroxaban group (*n*=118)	*P*
Slight bleeding (*n*)	35	32	0.352
Haematomas	10	12	
Subcutaneous ecchymosis	13	14	
Incision bleeding	12	6	
Major bleeding (*n*)	1	0	0.316
Incision bleeding time (min)	7.52 ± 1.32	6.19 ± 1.31	<0.001

## Discussion

Since DVT and correlated PE are difficult to prevent and the incidence after total hip or knee replacement reaches up to nearly 10%, DVT and PE are considered to be fatal complications [19]. The present study is supposed to estimate the effectiveness of rivaroxaban on preventing DVT after hip replacement for aged diabetic patients with femoral neck fractures. Our results investigated that rivaroxaban inhibited the formation of DVT after hip replacement for aged diabetic patients with femoral neck fractures.

After comparisons of TEG parameters and blood coagulation function during perioperative period between the rivaroxaban and control groups before and after operation, the outcomes indicated that rivaroxaban could lower the degree of blood hypercoagulability in diabetics. Rivaroxaban is named as a standard anticoagulant therapy for preventing and curing DVT for its effectiveness [22]. Compared with before operation, the rivaroxaban group has extended R time and K time, decreased α, MA and G and CI. TEG is considered to be a beneficial method to estimate coagulability [23]. K stands for time to clot forming and G represents clot strength [24]. The increase in time indicates the deficiency of coagulation and the MA stands for the biggest strength of coagulation. The larger the α, the faster the coagulation [25]. Derived as a discriminant analysis, CI distinguishes hypercoagulability by estimating the weights of different TEG values [26]. After operation, compared with the control group, the number of platelets and haemoglobin in the rivaroxaban group was significantly smaller and the PT and APPT obviously decreased. Natesirinilkul et al. investigated that a higher platelet count may lead to hypercoagulable state [27]. Coagulation tests are mostly conducted with PT and APPT [28]. Edoxaban, functioning as an anticoagulant like rivaroxaban, causes prolonged PT and APPT presenting a decreased rate of thrombin formation in a concentration-dependent way [29].

The study showed the incidence of DVT in the control group was significantly higher than that in the rivaroxaban group. No PE occurred in either of the two groups. Rivaroxaban is a newly developed oral anticoagulant therapy that is superior to other antagonists [30]. Owing to its hypothetical therapeutic potential and consistent *in vivo* antithrombotic activity among venous and arterial thrombosis models, rivaroxaban is believed to be a perfect anticoagulant agent providing great advantages to outpatient services [31]. According to Hillarp et al. [32], without laboratory monitoring, rivaroxaban is able to decrease the coagulation procedures with high efficiency and may inactivate FXa activities in some coagulation assays. Khalafallah et al. [33] investigated that in the treatment of damages in inferior vena cava caused by DVT, rivaroxaban alleviated the situation of oedema and DVT, while some standard anticoagulation therapies failed to take effect.

Logistic regression analysis showed that risk factors of DVT were BMI ≥25, abnormal coagulation indicators, the use of cemented femoral hip prosthesis, high haemoglobin content and non-ankle pump exercises after operation. In accordance with the findings here, one previous study showed the average BMI of DVT patients was higher than that of non-DVT patients to a large extent [34]. Abnormal coagulation indicators in patients with hip diseases may indicate raised risk factors causing thromboembolic phenomena [35]. In order to decrease secular complications, contemporary cementing techniques have been developed to mend the embedded femoral prosthesis in the medullary cavity [36]. People with haemoglobin above 12.5 g/l are believed to have higher risk of hypercoagulability [37,38]. Using a calf muscle pump pumping blood to the heart, ankle-pumping exercises are widely conducted to prevent DVT [39]. To conclude, we suggest that there should be further study to verify if rivaroxaban can be used for dilute blood. And a better understanding of the underlying mechanisms of rivaroxaban involved in DVT in case of hip replacements may control a situation potentially presenting in diabetic patients by acting in a preventive manner.

## Conclusion

To sum up, the present study demonstrated that rivaroxaban could be used to inhibit DVT after hip replacement for aged diabetic patients with femoral neck fracture. Although the current study was carried out with limited number of patients and further investigation is needed, it still provides therapeutic guidance for controlling the occurrence of DVT.

## Abbreviations

APTT: activated partial thromboplastin time
BMI: body mass index
CDDU: color duplex Doppler ultrasound
CI: coagulation index
CT: computed tomography
DVT: deep vein thrombosis
G: clot elasticity
K time: clot formation time
MA: maximum amplitude
PE: pulmonary embolism
PT: prothrombin time
R time: reaction time
TEG: thrombelastography
α: α angle
